# Rehabilitation of an Extremely Edentulous Atrophic Maxilla with a Pseudoskeletal Class III Relationship

**DOI:** 10.1155/2019/5696837

**Published:** 2019-04-21

**Authors:** Gregor-Georg Zafiropoulos, Tae Ho Yoon, Moosa Abuzayda

**Affiliations:** ^1^University of Sharjah, College of Dental Medicine, Sharjah, UAE; ^2^Hamdan Bin Mohammed College of Dental Medicine, Mohammed Bin Rashid University, Dubai, UAE

## Abstract

The skeletal class III relationship presents complex dentoalveolar problems, requiring multidisciplinary treatment. In edentulous people, severe atrophy of the jawbone simulates the clinical appearance of a skeletal class III relationship (pseudoskeletal class III), which presents major problems for rehabilitation. This article describes the rehabilitation of a 67-year-old patient with a pseudoskeletal class III relationship. The mandible was restored with two implant-supported bar-retained overdentures using clips for retention. The extremely atrophic maxilla was restored with a combination of sinus augmentation, implant placement, and classic prosthodontic treatment using an electroformed mesostructured overdenture with swivel lock attachments on an implant-supported bar. By performing minimal augmentative and implant surgeries and using the possibilities and advantages of classic prosthetic dentistry, the clinical situation described here could be managed and the atrophic maxilla could be rehabilitated.

## 1. Introduction

The prosthetic rehabilitation of a fully edentulous and atrophic arch with implant-retained dentures requires thorough planning and should not only provide the correct vertical height and maxillary-mandibular relationship but also be esthetically acceptable [1–4]. The use of implant-supported overdentures is a treatment option for the restoration of fully edentulous jaws when conventional dentures have deficient retention due to advanced bone atrophy [1–4]. In older complete denture wearers, advanced jawbone atrophy simulates the clinical situation of a skeletal class III relationship combined with vertical alveolar bone loss; this condition is classified as pseudoskeletal class III. The true skeletal class III condition presents complex dentoalveolar problems, resulting from maxillary retrognathism and mandibular prognathism [5]. In such cases, rehabilitation presents a major challenge and multidisciplinary treatment is necessary. The application of regenerative techniques to the soft and hard tissues, sinus augmentation, implant placement, and/or Le Fort I osteotomy (e.g., orthognathic surgery) is often required, necessitating the involvement of periodontists, prosthodontists, and orthodontists, as well as oral and maxillofacial surgeons [5–10]. In pseudoskeletal class III cases in aged persons, resulting from multiple tooth extractions and severe or advanced atrophy of the jawbone over time, rehabilitation is also a major problem due not only to high levels of discomfort in the surgical and postsurgical phases but also the reduced regenerative capacity of the soft and hard tissues in this population. For this reason, less-invasive procedures for the implant-supported restoration of severely atrophic jaws, such as the placement of tilted or pterygoid implants, have been introduced [6–8].

There is debate of how many implant support will be sufficient for the retention of fully edentulous mandibular prosthesis. Recently, it has been concluded that two implant removable overdentures should become the first choice of the treatment for the mandible supporting the McGill and York consensus statement [11–13].

This article describes the rehabilitation of an elderly patient with a pseudoskeletal class III relationship. In this case, the mandible was restored with two implant-supported bar retained overdentures using clips for retention, and the extremely atrophic maxilla was restored with a combination of implant placement, sinus augmentation, and classic prosthodontic treatment using an electroformed mesostructured overdenture with swivel lock attachments on an implant-supported bar.

## 2. Case

**Figure 1:**
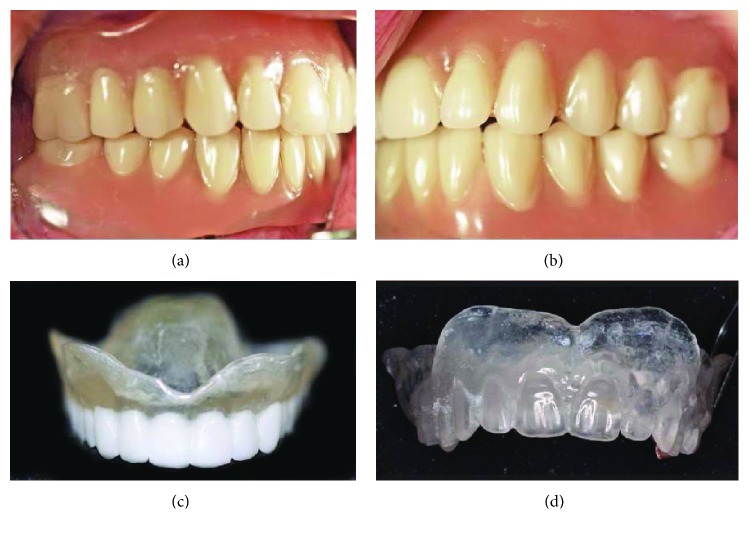
Initial examination: (a) patient's dentures, right side; (b) patient's dentures, left side; (c) DentDu-Brm for the implant planning using barium sulfate resin for the teeth; (d) DentDu fabricated from clear resin.

A 67-year-old nonsmoker female patient in good general health was referred for implant placement and prosthetic rehabilitation in March 2012. She was completely edentulous and had worn full dentures in both arches for 17 years. The dentures at the time of presentation had been fabricated 3 months previously, and the patient was satisfied with the extraoral appearance of the lips and face and tolerated the vertical dimension (Figures 1(a) and 1(b)). Her main complaint was the loss of denture retention even though she used denture adhesive daily. The patient did not grant permission for extraoral photography.

Two (2) duplicates of patient's maxillary dentures were fabricated. One (DentDu-Brm) for the implant planning using clear resin (Paladur; Heraeus Kulzer, Hanau, Germany) for the base and barium sulfate resin (Acryline DVT, Anaxdent, Stuttgart, Germany) for the teeth (Figure 1(c)). The second one was using the same clear resin for the base and the teeth in order to serve as a transfer guide during restoration (DentDu) (Figure 1(d)). Duplicates were checked for intraoral fit before use.

**Figure 2:**
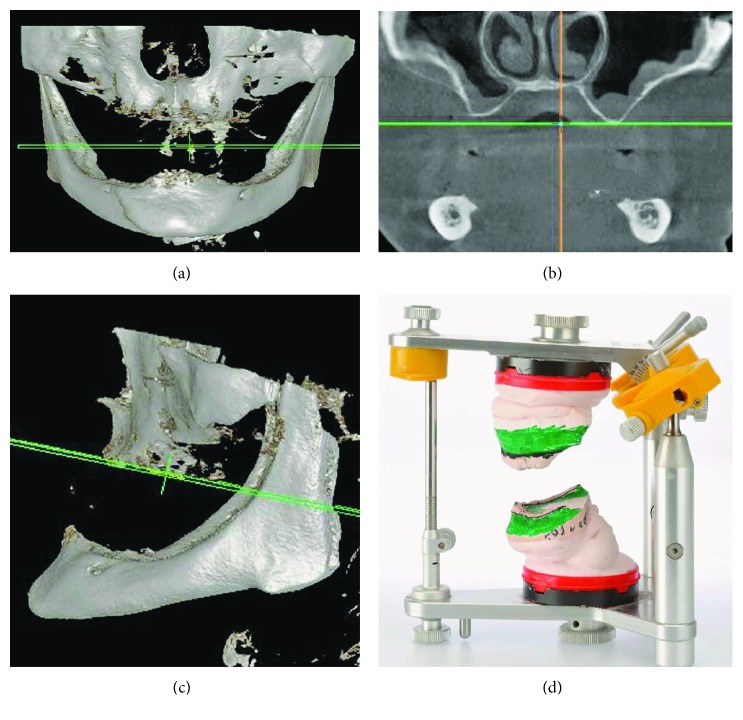
CBCT bone masking showing generalized advanced atrophy of the maxilla and posterior mandibular atrophy: (a) front view; (b) sinuses in sagittal section; (c) side view; (d) articulated casts show patient's jaw relationship.

Clinical examination and cone beam computed tomography (CBCT) revealed an extreme atrophy of the maxilla and mandible (Figures 2(a)–2(c)).

The patient's dentures were used for impressions with an alginate material (Alginat rosa; Omnident, Rodgau, Germany) and as guides for the articulation of the casts. Analysis of the articulated casts showed large vertical distances between the maxillary and mandibular alveolar crests (2.3–2.4 cm in the premolar/molar area, 1.9 cm in the anterior area) and a horizontal distance of 1.3 cm in the anterior area (Figure 2(d)). Therefore, rehabilitation with removable restorations was suggested. As the patient refused lateral and vertical augmentation, the decision was made to refabricate implant-retained removable dentures.

**Figure 3:**
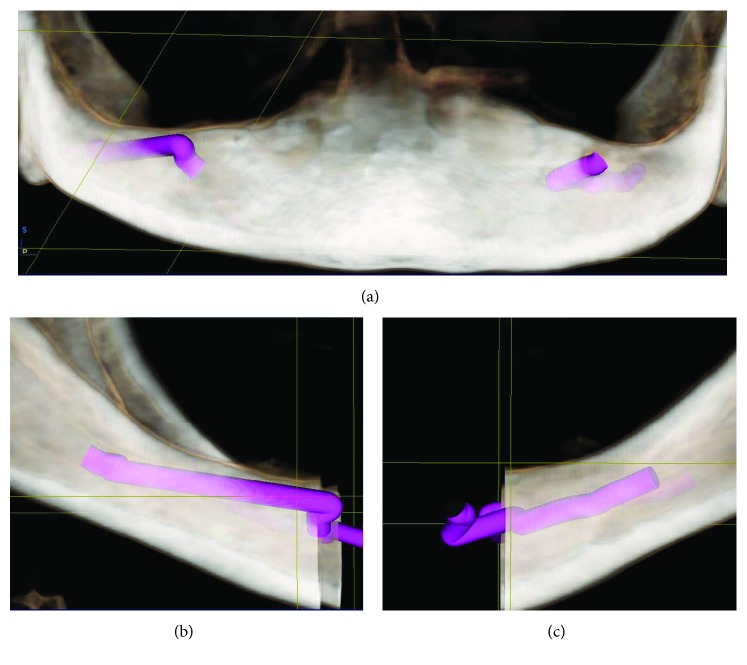
Tracking the path of inferior alveolar nerve and location of mental foramen for surgical implant placement: (a) frontal; (b) right side; (c) left side.

**Figure 4:**
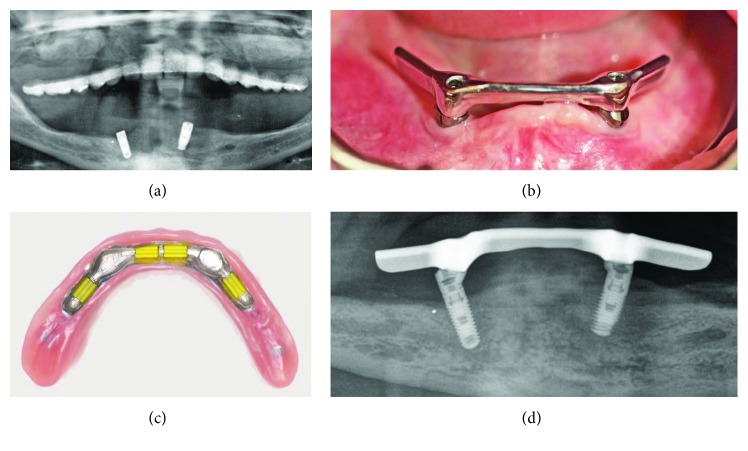
Sinus augmentation and mandibular restoration: (a) OPG after sinus augmentation and implant placement in the mandible; (b) implant-retained bar; (c) clips for retention of the base over the bar; (d) OPG section after loading of the mandibular implants.

Both sinuses were grafted using a bovine xenograph (CompactBoneB; Dentegris, Duisburg, Germany), and the lateral windows were covered with porcine collagen membranes (BoneProtectGuide; Dentegris). In the same session, based on the CBCT, two implants were placed in positions #22 and #27 (SB line, 3.75 mm × 11 mm, Dentegris) and loaded immediately with a bar-retained overdenture using system-specific, prefabricated, angled, and tapered abutments (Figure 3). A bar was milled from type 3 cobalt-chromium-molybdenum alloy (CoCrMo; ZENOTEC NP; Wieland, Pforzheim, Germany), and a metal base (housing) was cast with CoCrMo (Ankatit, Anka Guss, Waldaschaff, Germany). Elastic plastic clips (Preci Matrice; CEKA, Waregem, Belgium) were used to retain the base over the bar (Figure 4).

**Figure 5:**
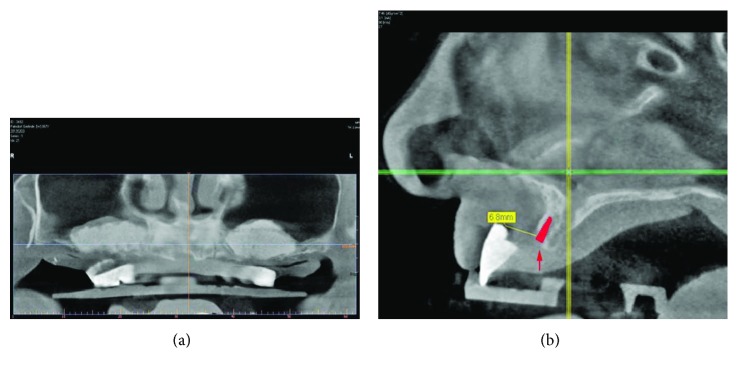
Implant planning. CBCT of the maxilla with DentDu-Brm *in situ* before implant placement: (a) sagittal section and (b) axial section.

Five months after sinus augmentation, a total of six implants (#3, 4, 6, 11, and 13; SB line; Dentegris) were placed. CBCT planning software (Sicat Implant, Sicat GmbH, Bonn, Germany) showed that the ideal tooth position was 6.8 mm in front of the alveolar ridge (Figure 5). However, one implant (left sinus, #11) could not be stabilized, so treatment proceeded with the five remaining implants.

**Figure 6:**
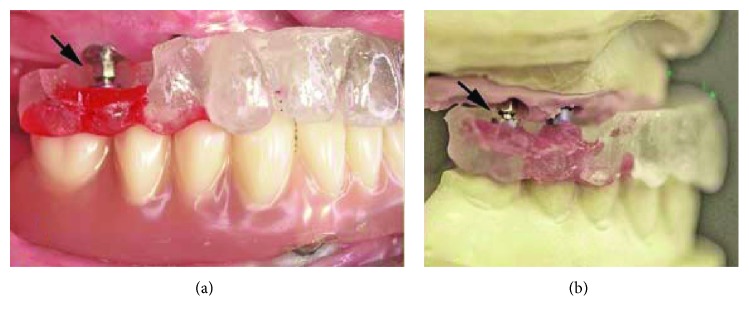
(a) DentDu retained on BATTs in occlusion and (b) articulated master cast with the BATTs and DentDu.

The maxillary implants were uncovered 6 months after placement, and impressions were made using an open-tray technique and a polyether impression material (Impregum Penta Soft; 3M ESPE, Neuss, Germany). System-specific ball attachments (BATTs) with their retention elements were mounted on three implants for laboratory transfer. Access windows were created on the buccal side of the DentDu in the areas of the BATTs, and the retention elements were fixed to the DentDu with self-curing modeling resin (Pattern Resin; GC, Alsip, IL, USA). Centric jaw relation was recorded using the same resin (Pattern Resin; GC America Inc., Alsip, IL, USA; Figure 6(a)). A master cast was fabricated, and the BATTs were mounted on the implant analogs. The DentDu with retention elements was then positioned on the master cast, and casts were articulated under the guidance of the DentDu and jaw record (Figure 6(b)).

**Figure 7:**
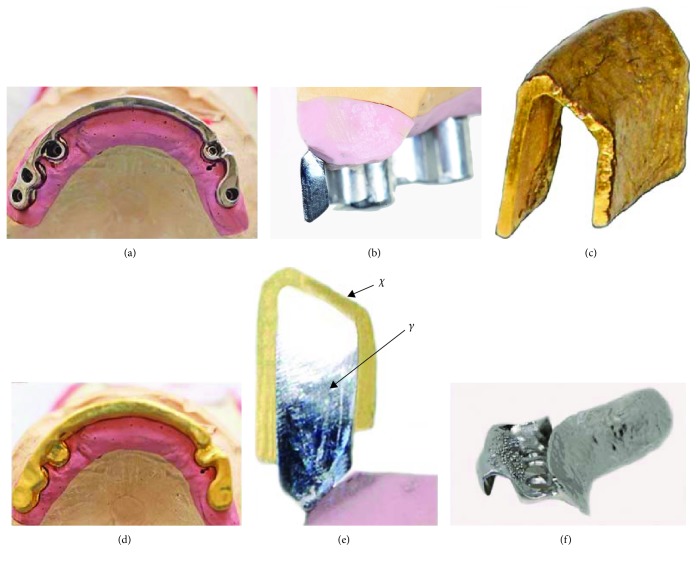
(a) The fabricated maxillary bar; (b) cross section of the bar on a model; (c) ELMES; (d) ELMES conforming to the shape of the bar; (e) cross section on a model demonstrating the use of the ELMES (*X*) over the bar (*Y*); (f) casted metal framework (laboratory work shown on a demonstration cast).

**Figure 8:**
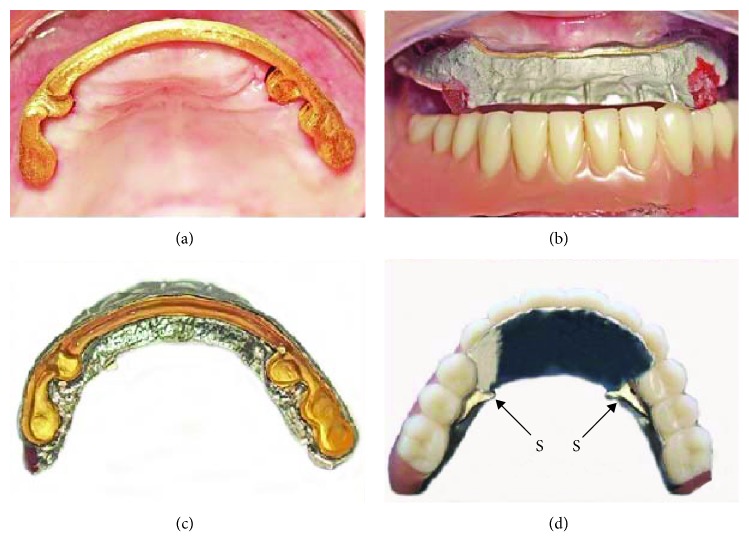
(a) ELMES try-in; (b) verification of the occlusal relationship with the framework in; (c) ELMES fixed in the framework; (d) external view of the metal framework (S: swivel-type lock attachments).

**Figure 9:**
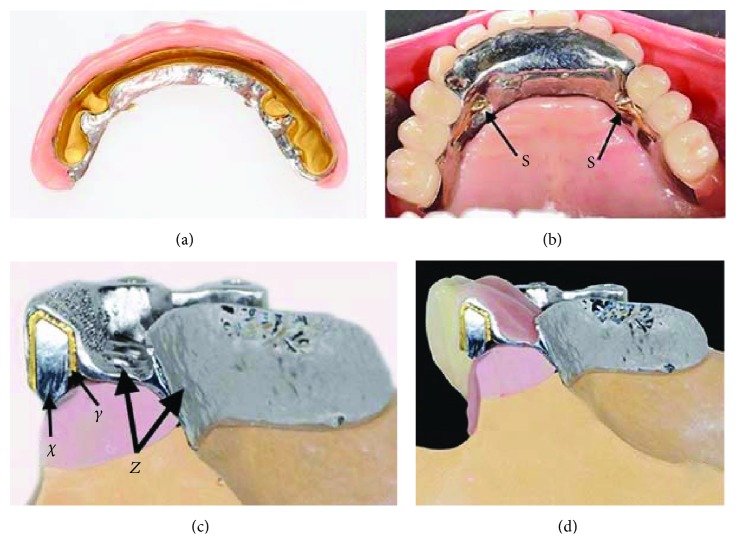
(a) Finished maxillary restoration inner side; (b) maxillary restoration *in situ* (S: swivel-type lock attachments); (c) cross section of the construction on a model: bar (*X*), ELMES (*Y*), and metal framework (*Z*); (d) the veneered construction. (a, d) Laboratory work shown on a demonstration cast.

The maxillary bar was designed using a superimposed DentDu and was positioned as far labial as possible to provide support for the denture base (Figures 7(a) and 7(b)). After verification of the bar's fit, an electroformed mesostructure (ELMES; Galvanogold, Wieland) with a thickness of 0.25 mm was fabricated to conform to the shape of the bar (Figures 7(c)–7(e)) [14, 15]. Framework was cast with CoCrMo alloy (Ankatit, Anka Guss, Waldaschaff, Germany) and tried in the patient's mouth to check its passive fit (Figure 7(f) and Figures 8(a) and 8(b)). In addition, a framework for an overdenture base was fabricated by casting CoCrMo alloy (Ankatit, Anka Guss; Figure 8 and Figures 9(a) and 9(b)). The U-shape framework covering the entire ridge including the lingual slope and partial palatal coverage provided rigidity and gains some degree of stability and support from the tissue. To provide passive retention and locking, two swivel-type lock attachments (swivel-type lock, Heraeus Kulzer, Hanau, Germany) were cast with a gold alloy (Hera; Heraeus Kulzer) (Figure 8(d) and Figures 9(c) and 9(d)) [16].

**Figure 10:**
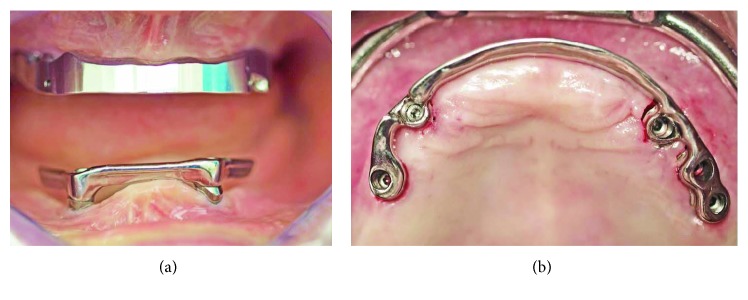
Bars mounted onto the implants: (a) front view and (b) palatal view.

**Figure 11:**
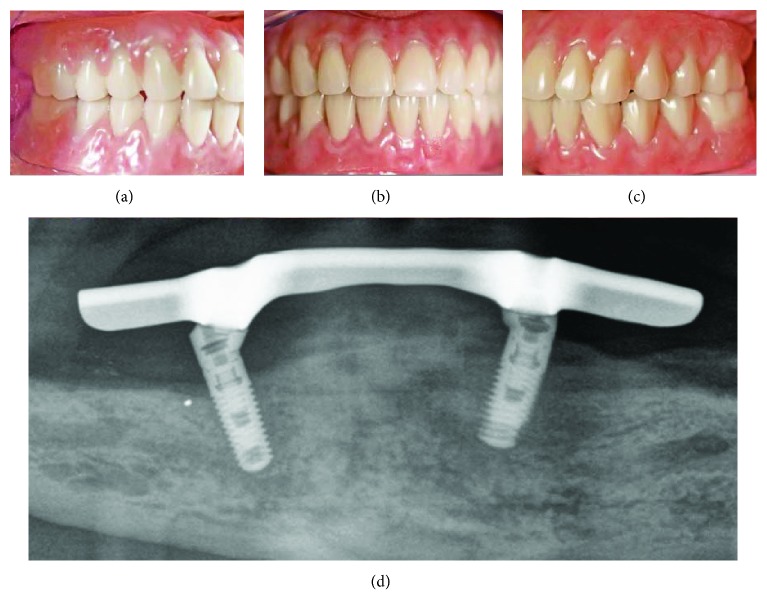
Final restorations in situ: (a) right; (b) front; (c) left; (d) OPG 3 years after treatment.

**Figure 12:**
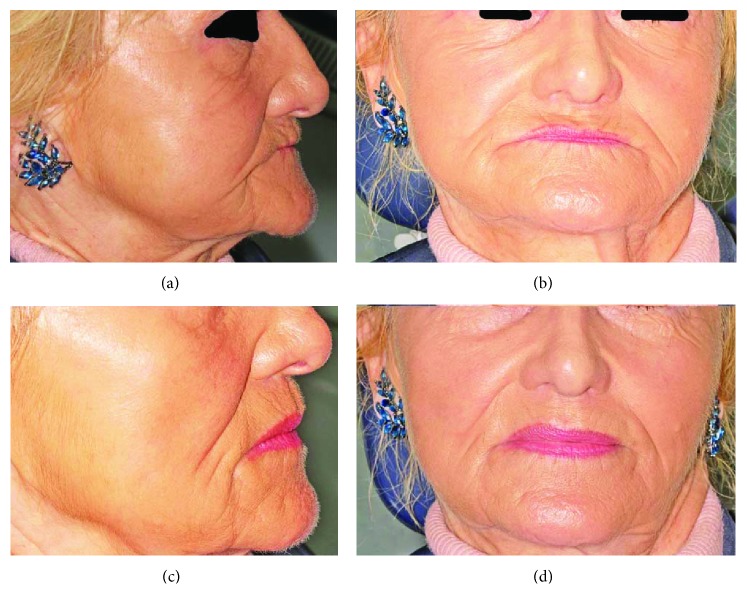
Final restoration delivered: (a) before treatment (lateral); (b) before treatment (frontal); (c) after treatment (lateral); (d) after treatment (frontal).

The fabricated maxillary bar was mounted on the implants, the ELMES was placed on the bar (Figure 8(b)), and the jaw relationship was verified with the framework in place (Figure 8(c)). Subsequently, the ELMES was fixed to the framework using a self-cure compomer (AGC Cem, Wieland; Figure 9(c)). Denture teeth (SR; Ivoclar Vivadent, Ellwangen, Germany) were set and tried in before the finishing process. The denture was finished with the use of an autopolymerizing acrylic resin base material (PalaXpress Ultra; Heraeus Kulzer; Germany) and delivered back to the office on the same day (Figures 10–12) [1–4, 17, 18].

The patient was then enrolled in a 6-month maintenance program. During the three years of observation period, at the follow-up visits, oral hygiene was reinforced together with debridement and prophylaxis.

## 3. Discussion

For patients with extensive residual ridge resorption, the replacement of hard and soft tissues with a removable denture may be a more suitable option than the use of a cemented (or fixed type) restoration [1–4]. The analysis and management of the restorative space in edentulous patients with implant overdentures are important for the planning of implant placement and for the design of the denture [16, 19, 20]. To retain dentures in patients who have lost large segments of alveolar ridge, the use of clips or electroformed copings on the bars, horizontal path bar attachment systems, and locking attachment systems has been reported [5, 6, 14–22].

In treatments involving removable dentures, decisions about tooth length and position are critical because they affect esthetics and function. In the case described here, the patient accepted the profile and appearance of the original denture, and a duplication of this denture was used as a guide for new denture fabrication in terms of tooth position and tissue and lip support, as well as for the CBCT-based implant placement plan and the design of overdenture substructures. As a general rule, OPGs are made at initial examination in order to assess the situation and after sinus augmentation. In the case presented here, we used a CBCT at the initial examination. The reason was the advanced atrophy on clinical examination; 3D imaging provided more accurate assessment of the sinuses and the existing bone.

It is recommended to utilize the dental implants as a means for improving retention in cases of poor ridge support and its retention would be compromised. Only two implant placements were possible in this severely resorbed mandibular ridge, and their primary stability was good that additional augmentation to place more implants was not performed. The mandibular arch was restored with an implant-supported bar-retained overdenture using clips for retention, which is a treatment option for edentulous jaws because of the high implant/prosthesis survival rate and limited incidence of technical complications [23–28].

The quality and longevity of implant-supported removable dentures are affected by the passive fit of the superstructure. In the case presented here, ELMES used in the maxillary restoration allows for precise fitting of dental restorations and is particularly useful to achieve passive fit because it enables tension-free placement of the framework onto the abutments [5, 6, 17, 22, 29]. Furthermore, the palatal edge of the prosthesis remained in contact with the mucosa as the palate did not appear to resorb to any appreciable degree.

In the case described here, an implant-supported bar was placed buccally to the ridge crest to support the denture base and teeth, an ELMES was fabricated over it, and a U-shaped metal framework for the denture was designed to rest on the ridge crest and extend palatal to provide support and resistance with rigidity. This approach also strengthened the denture base and relieved the biting forces generated anteriorly by distributing stresses to the metal base. The swivel lock attachment provided rigid fixation on the implant-retained bar with minimal strain, preventing rocking movements of the denture during function. Therefore, this fixed-detachable denture with a swivel lock attachment system provided the comfort and assurance of a fixed implant-supported denture, as well as improved efficacy of masticatory function [16].

In the first three weeks following the delivery of the dentures, the patient learned to actuate the swivel lock attachment. It is reported from the systematic review that overdenture with two implants and with bar attachment has the least number of complications [30], and during the three years of follow-up period, no biological or prosthetic complications were observed.

Patient reported a high level of satisfaction with the dentures and an improved quality of life [31, 32].

A disadvantage of this approach is the initially higher laboratory cost due to the need for fabrication of multiple components. However, this cost may be offset by the need for fewer repairs and reduced patient discomfort. Furthermore, the application of the described technique, which combines surgical approaches with classic prosthetic treatment options, improves quality of life and avoids the need for more invasive surgery in elderly patients.
